# A Comparative Analysis of Functional Recovery in Surgical Rotator Cuff Tear Repair: Mini-Open Versus All-Arthroscopic Techniques

**DOI:** 10.7759/cureus.57529

**Published:** 2024-04-03

**Authors:** Dhruv Sharma, Mohit Tolani, Sohilkhan R Pathan, Sanjay Soni, Dhruv R Patel, Manan R Shroff

**Affiliations:** 1 Orthopaedic Surgery, Shree Krishna Hospital and Medical Research Centre, Pramukhswami Medical College, Bhaikaka University, Anand, IND; 2 Clinical Research Services (CRS), Bhanubhai and Madhuben Patel Cardiac Centre, Shree Krishna Hospital and Medical Research Centre, Anand, IND

**Keywords:** functional outcomes, all-arthroscopic technique, mini-open technique, surgical repair, rotator cuff tear

## Abstract

Introduction: Rotator cuff tears frequently lead to shoulder pain and impaired function, often necessitating surgical intervention to achieve the best results. The choice between mini-open and all-arthroscopic techniques remains a subject of debate, with each approach offering unique advantages and challenges. This study seeks to evaluate and compare the functional outcomes of surgical repair utilizing these two techniques, offering valuable insights into their relative effectiveness.

Material and methods: This retrospective observational study was conducted at Shree Krishna Hospital, Karamsad, involving patients treated surgically for rotator cuff tears over the past five years. Clinical records were reviewed to identify patients who underwent either mini-open or all-arthroscopic repair. Follow-up assessments were conducted using the Quick Disabilities of the Arm, Shoulder, and Hand (Quick DASH) score and the Visual Analog Scale (VAS) for pain. A statistical analysis was performed to compare outcomes between the two groups.

Results: A total of 33 patients were included, with 16 undergoing mini-open repair and 17 undergoing all-arthroscopic repair. The mean follow-up duration was 31.06 months for mini-open repair and 20.4 months for all-arthroscopic repair. No statistically significant variances were observed in the postoperative Quick Disabilities of the Arm, Shoulder, and Hand (Quick DASH) scores or Visual Analog Scale (VAS) scores between the two groups. Both techniques demonstrated satisfactory functional recovery and pain relief at long-term follow-up.

Conclusion: Our study provides evidence of comparable outcomes between mini-open and all-arthroscopic techniques for rotator cuff repair. Despite limitations such as a small sample size and the subjective nature of Quick DASH scores, both approaches offer promising results in terms of functional improvement and pain reduction. Further research is needed to assess short-term outcomes, cost-effectiveness, and patient satisfaction, but our findings support the continued use of both techniques in clinical practice.

## Introduction

Over a century ago, Codman first described the repair of the rotator cuff [1]. Rotator cuff lesions represent one of the more common incapacitating conditions of the shoulder. Although rotator cuff tears may be asymptomatic, chronic shoulder pain with debilitating shoulder function may occur. Rotator cuff tears usually occur in people aged 40 and over. On occasion, young throwing athletes sustain rotator cuff tears from trauma, instability, or impingement. Most physicians advocate nonoperative management for rotator cuff tears initially and reserve surgery as a secondary treatment. Surgical management for full-thickness rotator cuff tears remains a topic for debate, especially when the tear is extensive. Modalities for rotator cuff tear management include conservative options like rest, physical therapy, and medications. Surgical interventions such as open repair, mini-open repair, and arthroscopic repair are considered when conservative methods fail or for extensive tears. Each approach has its benefits and should be chosen based on factors like tear size, patient age, and surgeon expertise. One of the current procedures, mini-open (MO) repair, has been regarded as the gold standard for rotator cuff tear repair for decades; it has been proven to achieve good to excellent results in 90% of patients [2-5]. Due to its stronger suture fixation and shorter learning curve, the repair of the rotator cuff has been the first choice for many surgeons [6-8]. Over the past decade, with advances in surgical instruments and operative techniques, there has been a shift from MO to an all-arthroscopic (AA) technique in rotator cuff repair surgery. Faster recovery and better cosmetic results have led many surgeons to prefer the arthroscopic approach [9,10]. Despite both techniques being associated with good clinical outcomes, the determination of the most effective method of repair remains unresolved. This uncertainty persists despite the repair of the rotator cuff being the preferred option for many surgeons, primarily due to its stronger suture fixation and shorter learning curve [11,12].

Previous comparisons between the two approaches have highlighted distinct advantages for each. The mini-open (MO) procedure, for instance, is often noted for its smaller skin incisions, reduced soft tissue dissection, and lower likelihood of deltoid muscle detachment. Conversely, the all-arthroscopic (AA) procedure has been associated with benefits such as reduced postoperative pain, minimal deltoid morbidity, shorter hospital stays, and accelerated rehabilitation. Despite these observed differences, both techniques have demonstrated favorable clinical outcomes, leaving the optimal choice of approach still subject to debate within the medical community [13-17]. 

Several systematic reviews and meta-analyses conducted years earlier have consistently reported no significant differences between arthroscopic and mini-open (MO) repairs. These comprehensive analyses, spanning various studies and patient populations, have failed to identify the clear superiority of one technique over the other in terms of functional outcomes, complication rates, or long-term success rates. Despite the inherent differences in surgical approach and associated technical considerations, the collective body of evidence suggests that both arthroscopic and mini-open repairs offer comparable efficacy in addressing rotator cuff tears. However, the ongoing evolution of surgical techniques and advancements in technology may warrant revisiting these comparisons to ascertain if any emerging trends or refinements have influenced the relative merits of each approach [18].

Despite numerous studies comparing surgical techniques for rotator cuff tear repair, there remains a gap in the literature regarding the comprehensive evaluation of short- and long-term outcomes for both mini-open (MO) and all-arthroscopic (AA) approaches. In light of this gap, we have formulated a hypothesis positing that the all-arthroscopic (AA) procedure may yield superior clinical outcomes compared to the mini-open (MO) technique. By systematically assessing the early and late clinical outcomes of both AA and MO repair techniques, our study aims to provide valuable insights into the comparative effectiveness of these two commonly employed surgical approaches. Through this endeavor, we aspire to facilitate informed decision-making among surgeons and enhance patient care in the management of rotator cuff tears.

## Materials and methods

In this study, we conducted a comparative analysis of the outcomes between the all-arthroscopic and mini-open approaches for rotator cuff tear repair, focusing on mid-term and long-term follow-ups.

Data for this retrospective observational study were collected from a single center, Shree Krishna Hospital in Karamsad. The clinical records department was tasked with retrieving data from patients who had undergone surgical treatment for rotator cuff tears within the past five years. Operative notes of these patients were thoroughly reviewed to identify those who had been treated using either the mini-open or all-arthroscopic technique. Subsequently, all identified patients were contacted via telephone and scheduled for follow-up appointments in the outpatient department (OPD).

Patients meeting the following inclusion criteria were included: a) a diagnosis of a rotator cuff tear, b) individuals who underwent surgical treatment for a rotator cuff tear using either mini-open or all arthroscopic techniques, and c) available data on preoperative and postoperative Quick Disabilities of the Arm, Shoulder, and Hand (Quick DASH) scores and Visual Analog Scale (VAS) for pain.

The criteria for excluding a patient from this study were: a) incomplete medical records, b) individuals who underwent surgical treatment for rotator cuff tear using other techniques, c) comorbidities affecting shoulder function or outcomes assessment, and d) missing data on preoperative and postoperative Quick Disabilities of the Arm, Shoulder, and Hand (Quick DASH) scores and Visual Analog Scale (VAS) for pain.

For assessing functional outcomes, we utilized the Quick DASH score, a patient-reported outcome measure (PROM) known for its high construct validity and responsiveness in various upper limb pathologies. Upon obtaining consent, patients were provided with handouts containing the Quick DASH score questionnaire during their follow-up appointments. Their responses were recorded and compared with their preoperative Quick DASH scores retrieved from the clinical records.

Data analyses were performed using the statistical software STATA Version 14.2 (© 199-2024 StatCorp LLC.). Data were summarized using descriptive statistics (counts, percentages, mean, and range). We used an independent sample t-test at a level of significance (alpha) of 0.05 to compare the DASH score and VAS score between two surgical approaches (mini-open repairs versus all-arthroscopic repairs).

## Results

In our study, a total of 33 patients diagnosed with rotator cuff tears were enrolled, with 16 (48.48%) undergoing mini-open repair and 17 (51.51%) undergoing all-arthroscopic repair procedures. Among the patients who underwent mini-open repair, nine (56.25%) were females, and seven (43.75%) were males, while among those who underwent all-arthroscopic repair, six (35.29%) were males and 11 (64.70%) were females.

During the follow-up assessments, which occurred at varying intervals, the functional outcomes were evaluated using the Quick Disabilities of the Arm, Shoulder, and Hand (Quick DASH) score, and the pain intensity was assessed using the Visual Analog Scale (VAS). The mean follow-up duration for patients who underwent mini-open repair was 31.06 months (range: 6-46), whereas for those who underwent all-arthroscopic repair, it was 20.4 months (range: 6-45).

Before surgery, the mean Quick DASH score was comparable between the two groups, with patients undergoing mini-open repair scoring an average of 45.78 (range: 43.2-50) and those undergoing all-arthroscopic repair scoring 45.87 (range: 43.2-50). Following surgery, the mean postoperative Quick DASH score for patients who underwent mini-open repair significantly improved to 3.02 (range: 0-9.1), while for those who underwent all-arthroscopic repair, it remained similar at 45.87 (range: 43.2-50).

Regarding pain intensity, the mean preoperative VAS score for all patients was 6.4 (range: 5-8). At the follow-up assessments, the mean postoperative VAS score for patients who underwent all-arthroscopic repair significantly decreased to 1.2 (range: 0-4), whereas for those who underwent mini-open repair, it was 1.6 (range: 0-4) (Table 1).

**Table 1:** Comparison of patient demographics and surgical outcomes between mini-open and all-arthroscopic techniques for rotator cuff repair

Surgical Approach	No. of Male Patients (N)	No. Female Patients (N)	Mean Follow-up Duration (in months), Mean (Range)	Mean Preoperative Quick DASH Score, Mean (Range)	Mean Postoperative Quick DASH Score, Mean (Range)	Mean Preoperative VAS Score, Mean (Range)	Mean Postoperative VAS Score, Mean (Range)
Mini-Open	7	9	31.06 (6-46)	45.78 (43.2-50)	3.02 (0-9.1)	6.4 (5-8)	1.6 (0-4)
All-Arthroscopic	6	11	20.4 (6-45)	45.87 (43.2-50)	45.87 (43.2-50)	6.4 (5-8)	1.2 (0-4)

Statistical analysis using the Independent Samples t-test revealed no statistically significant difference in postoperative VAS or Quick DASH scores between the two surgical groups.

**Figure 1:**
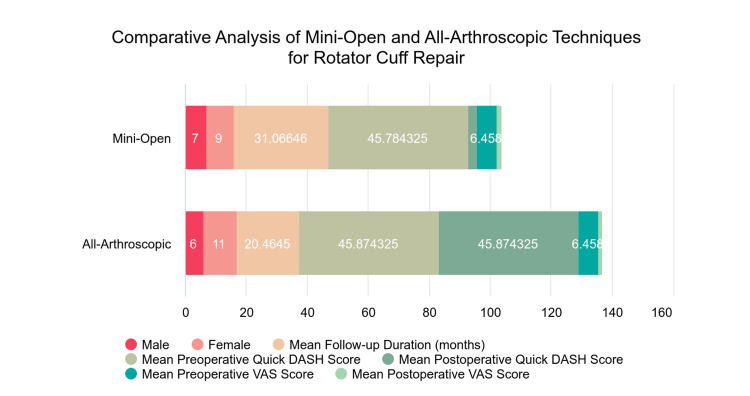
Comparative analysis of mini-open and all-arthroscopic techniques for rotator cuff repair Note: males and females are counts (N).

These results indicate that both the mini-open and all-arthroscopic repair techniques demonstrate similar effectiveness in enhancing functional outcomes and alleviating pain for individuals with rotator cuff tears. Nonetheless, additional long-term investigations involving larger cohorts are necessary to validate these findings and clarify any distinctions between the two methods (Figure 1).

## Discussion

It's worth noting that the majority of studies included in systematic reviews and meta-analyses were characterized by low methodological quality. Many of them lacked randomization in sample selection, and follow-up assessments were predominantly retrospective. This inherent limitation raises concerns regarding the reliability and generalizability of their findings. Despite the extensive volume of literature comparing arthroscopic and mini-open (MO) repairs, the scarcity of high-quality randomized controlled trials (RCTs) is notable. Nevertheless, the few RCTs that have been conducted offer valuable insights into the comparative effectiveness of these surgical approaches. Some of these studies have reported advantages associated with the arthroscopic technique, including shorter surgery times and improved range of motion at the end of follow-up. Additionally, arthroscopic repairs have been associated with reduced pain levels during the midterm period of postoperative rehabilitation compared to mini-open repairs. While these findings provide intriguing glimpses into the potential benefits of the arthroscopic approach, the limited number of RCTs underscores the need for further well-designed studies to validate and expand upon these observations [19,20].

Both mini-open and all-arthroscopic techniques for surgical repair of rotator cuff tears have demonstrated promising results in our study, suggesting that they can be considered standard treatments for this condition. However, it is important to acknowledge the distinct advantages and challenges associated with each approach. Mini-open repair of the rotator cuff offers the advantage of requiring minimal infrastructure support and has a relatively more forgiving learning curve compared to the all-arthroscopic technique. This makes it a viable option in settings where resources may be limited or where surgeons are still gaining experience in shoulder surgeries. On the other hand, the all-arthroscopic technique demands proper instrumentation, a well-trained surgical team, and an experienced surgeon due to its more technically demanding nature and steeper learning curve. Despite these differences, both techniques have been shown to provide excellent postoperative outcomes at long-term follow-up. This suggests that regardless of the approach chosen, patients can expect satisfactory functional recovery and pain relief over time. The choice between mini-open and all-arthroscopic repair should be guided by considerations such as the surgeon's proficiency, patient preferences, resource availability, and the particular attributes of the rotator cuff tear.

When contrasting our study with the randomized controlled trial carried out by van der Zwaal et al., both studies were designed to evaluate the clinical outcomes associated with all-arthroscopic (AA) versus mini-open (MO) techniques for rotator cuff repair. While our study included 33 patients with a minimum one-year follow-up duration, van der Zwaal et al. analyzed 95 patients with a one-year follow-up. Both studies observed improvements in primary and secondary outcome measures postoperatively, with no statistically significant differences in overall mean postoperative outcome scores between the AA and MO groups. On the other hand, van der Zwaal et al. observed notably superior enhancements in DASH score, VAS pain, and impairment, as well as active forward flexion, at the six-week follow-up among participants in the all-arthroscopic (AA) group compared to those in the mini-open (MO) group. Additionally, similar rates of complications, including retears and adhesive capsulitis, were observed between the two surgical approaches in both studies. These findings contribute valuable insights into the comparative effectiveness of AA and MO repair techniques for rotator cuff tears, aiding clinicians in decision-making for patient management [20].

When comparing our study to Liu et al.'s randomized clinical trial, it's important to note that both studies assessed the outcomes of all-arthroscopic (AA) versus mini-open (MO) repair techniques for rotator cuff tears. While our study included patients with a minimum one-year follow-up duration, Liu et al. reported an average follow-up duration of 16.6 months. Both studies found longer operative times for the all-arthroscopic approach compared to mini-open repair, reflecting the increased technical demands of the former. Although Liu et al. observed significant differences in the range of motion, pain scores, and functional outcomes between the two groups at various time points, our study did not find significant differences in postoperative outcomes between the AA and MO groups. Similarly, both studies reported comparable rates of complications between the two surgical approaches. These findings collectively contribute to the understanding of the comparative effectiveness and safety of AA and MO repair techniques for rotator cuff tears, aiding clinicians in decision-making for patient management [21].

Limitations of the study include the relatively small sample size and the subjective nature of responses obtained through the Quick Disabilities of the Arm, Shoulder, and Hand (Quick DASH) score. Furthermore, our study focused primarily on mid-term and long-term outcomes, which may limit the generalizability of our findings to short-term outcomes. Future research should aim to assess the comparative effectiveness of these techniques in the short term, as well as explore their cost-effectiveness and patient satisfaction. Additionally, ongoing advancements in surgical technology and techniques may lead to further refinements and improvements in outcomes for patients undergoing rotator cuff repair, warranting continued investigation in this area.

## Conclusions

In conclusion, our study provides valuable insights into the comparative effectiveness of mini-open and all-arthroscopic techniques for rotator cuff repair. Despite limitations such as a small sample size and the subjective nature of Quick DASH score responses, our findings suggest comparable outcomes between the two approaches in terms of functional recovery and pain relief at mid-term and long-term follow-up. Further research is warranted to assess short-term outcomes, cost-effectiveness, and patient satisfaction. Our study underscores the importance of ongoing advancements in surgical technology and techniques in refining outcomes for patients undergoing rotator cuff repair. These findings contribute to the evidence base guiding clinical decision-making and patient management in this field.
